# Implementation of rural telehealth physical therapy for chronic low back pain: qualitative findings informing a randomized clinical trial aimed at decreasing opioid use

**DOI:** 10.3389/fresc.2026.1951507

**Published:** 2026-10-27

**Authors:** Kisha J. Ali, Kevin H. McLaughlin, LaPricia L. Boyér, Gabrielle Martino, Richard L. Skolasky

**Affiliations:** 1 MedStar Health Research Institute, Columbia, MD, United States; 2 Department of Physical Medicine and Rehabilitation, The Johns Hopkins University School of Medicine, Baltimore, MD, United States; 3 Department of Orthopaedic Surgery, The Johns Hopkins University School of Medicine, Baltimore, MD, United States

**Keywords:** back pain, implementation, opioid, rural health, telehealth

## Abstract

**Background:**

Low back pain (LBP), the most expensive health condition in the United States, accounts for over $134.5 billion in federal spending. As a leading cause of disability worldwide, LBP is a common reason for initiating prescription opioid therapy and often requires ongoing multidisciplinary care. In rural settings, where provider shortages, transportation barriers, and geographic constraints limit access to preventive and specialty healthcare for approximately 60 million Americans, telehealth can bring access to nonpharmacologic treatments of LBP, such as physical therapy, to patients. A qualitative analysis was conducted as part of the ARBOR-Telehealth study, a type 1 hybrid effectiveness-implementation of a pragmatic clinical trial, to evaluate a telehealth-delivered physical therapy intervention for adults with chronic LBP in rural Maryland.

**Methods:**

In partnership with TidalHealth, a rural health system, nine individuals (patients, physical therapists, rural stakeholders) participated in pre-implementation, semi-structured, baseline video interviews. For methodological rigor, qualitative data were analyzed adhering to the trustworthiness parallel to quantitative data.

**Results:**

Ten overarching themes (access to care, technology, age-related concerns, usefulness, broadband internet, comparison to in-person care, equipment, space, acceptance of telehealth, therapeutic bonds) illuminated challenges that could affect patient engagement and facilitated the co-design of actionable mitigation strategies for implementation.

**Conclusions:**

This study revealed context-specific insights on the feasibility of delivering guideline-based LBP physical therapy via telehealth in rural communities. It contributes evidence to ongoing National Institutes of Health efforts focused on improving pain management and reducing reliance on opioids in communities disproportionately affected by the overdose crisis.

## 1 Introduction

Rural populations across the United States (U.S.) face significant and well-documented health disparities compared with urban and suburban populations. Approximately 60 million Americans, nearly one in five, live in rural areas (1), where geographic isolation, healthcare workforce shortages, and constrained infrastructure limit access to preventive, specialty, and ongoing healthcare (2). These rural health challenges are often associated with a higher burden of chronic conditions (3) and poorer overall health outcomes among rural populations (4), including exacerbation of conditions such as low back pain (LBP) (5), diabetes (6), cardiovascular disease (7), and opioid use disorder (8–10). Among these conditions, chronic musculoskeletal pain presents substantial challenges for populations and, in the context of rurality, poses an even higher concern (11).

Low back pain (LBP) is a leading cause of disability worldwide and the most expensive health condition in the U.S., accounting for over $134.5 billion in spending per year (12). LBP is also among the most common reasons for initiating prescription opioid therapy (13, 14). LBP often requires ongoing multidisciplinary care that can be difficult to access in rural settings because of specialty provider shortages, travel and transportation burdens, and geographic isolation (8). Telehealth represents a promising avenue for expanding healthcare access to nonpharmacologic treatments of LBP, such as physical therapy, within the rural care delivery landscape. It brings guideline-based care closer to rural patients, creating new pathways for managing pain more effectively (11, 15, 16). Broadening patient access to physical therapy through telehealth offers a valuable opportunity to support the management of LBP, potentially reducing the reliance on opioid medications for treatment (3, 17–19). Advancing telehealth models to treat LBP aligns with U.S. priorities to improve care for chronic pain, and contributes to ongoing national efforts associated with reducing opioid dependence, particularly in rural communities, creating new pathways for managing pain more effectively (10, 20, 21).

The ARBOR-Telehealth study was funded by the National Institutes of Health (NIH) as part of its Helping to End Addiction Long-term (HEAL) initiative, in a nationwide effort to expedite scientific solutions that stem the public health opioid crisis (22–24). TidalHealth, a rural community health system in Maryland, served as partners. Patient enrollment for the ARBOR-Telehealth study began in January 2025 and continues until October 2027. The study will compare the effectiveness of two interventions, physical therapy delivered via real-time telehealth visits and a web-based educational program, in reducing LBP-related disability and opioid use among patients with chronic LBP in rural settings. The parent ARBOR-Telehealth randomized clinical trial evaluates risk-informed physical therapy delivered by video, emphasizing exercise, education, and self-management; detailed intervention and eligibility procedures are described in the published protocol (23). Rurality is defined using the Rural-Urban Commuting Area (RUCA) classification system developed by the U.S. Department of Agriculture (USDA) and the U.S. Census Bureau, using zip codes to dichotomize rural and non-rural areas (25). A further outcome of this work is to expand access to guideline-adherent, non-pharmacologic care for individuals living with chronic LBP in rural settings (15, 24, 26). Therefore, the study emphasizes a need for establishing a sustainable telehealth care-delivery model that can continue being implemented post-study, and scaled for use in other rural communities if found to be effective (13, 16, 27, 28).

The successful implementation of rural healthcare innovations relies on more than clinical effectiveness (29, 30). Structural and behavioral impediments, including technology access, health literacy, mistrust in remote care, and workforce deficits, frequently inhibit the real-world uptake of valuable healthcare interventions (31, 32). Such contextual challenges repeatedly highlight the importance of pre-implementation evaluations, which serve to identify critical local conditions necessary for early adoption, allowing for mitigation strategies to be embedded before starting an intervention (32–34). This evaluation provided such data.

This qualitative evaluation component of the ARBOR-Telehealth study focused on understanding contextual factors that could guide planned implementation of telehealth-delivered physical therapy for LBP in rural settings (35). The parent randomized trial's design and intervention procedures are described in the published protocol (23). Although telehealth barriers and facilitators are well described, detailed reporting of how stakeholder engagement informs implementation remains limited (35). This qualitative component provides a context-specific account of how perspectives from patients, providers, and rural-health stakeholders collectively informed implementation planning in rural Maryland that could help guide a more effective implementation of telehealth for LBP in rural settings (36). These data can facilitate the successful implementation of remote telehealth physical therapy by offering insights on patient engagement, feasibility, acceptability, and use and utility, as well as barriers and facilitators to care delivery. If not considered, local context may negatively influence study impact and outcomes, as many avoidable, small failures can accumulate. The data offer guidance for tailoring similar telehealth interventions to the unique needs of rural populations and rural healthcare delivery. It also informs broader national efforts to expand and scale accessible rehabilitative care through telehealth. The findings contribute to the emerging body of evidence supporting implementation planning for telehealth-delivered physical therapy for LBP in rural healthcare settings.

## 2 Materials and methods

### 2.1 Assessment timeframe

Qualitative data were collected as part of a baseline assessment in June 2024 to inform planned implementation starting January 2025.

### 2.2 Study population

Nine individuals associated with TidalHealth participated in baseline interviews to inform pre-implementation planning. Participants were eligible if they held roles relevant to the design, delivery, or receipt of the intervention. Participants were excluded if they were younger than 18 years or were neither receiving care at, nor working for, TidalHealth. A purposive sample of participants comprising patients with chronic LBP living in rural Maryland (*n* = 3), physical therapists and primary care physicians (*n* = 4), and Maryland Public Health Association and Maryland Rural Health Association stakeholders (*n* = 2). Patients were identified from a registry of rural individuals living with chronic LBP who had previously agreed to participate in future back pain-related research. Physical therapists were provided by TidalHealth. Stakeholders were selected based on their involvement in rural health advocacy. Potential participants were approached through TidalHealth. Invitation and decline counts were not recorded, and differences between participants and those who declined could not be assessed. Rurality was defined using the USDA/Census RUCA classification system (25). Demographic characteristics were not collected because these interviews focused on role-specific contextual perspectives for implementation planning. Patients, providers, and rural-health stakeholders contributed perspectives relevant to the receipt, delivery, and local organization of care. The nine interviews were considered collectively to obtain complementary perspectives on the receipt, delivery, and organization of care. The analysis considered recurring ideas among participants regardless of role, alongside role-specific perspectives. The purpose was to develop an overarching understanding of anticipated implementation issues and inform practical adaptations; thematic saturation was not an objective (37).

### 2.3 Data collection

Qualitative interviews were conducted via secure video conferencing to accommodate geographic distance, and informed consent was obtained at the start of the interview. Interviews were conducted using a semi-structured interview guide developed by the study team to ensure consistency across respondent groups. Guide development was informed by study team discussions and relevant literature, with questions aligned to the contextual information needed for implementation planning. The guide featured open-ended questions that moved from general to specific topics and was designed to elicit stakeholder perceptions of the telehealth use, potential barriers and facilitators of use, suggestions on optimizing implementation, and improving the remote delivery of physical therapy care. Interviews were audio-recorded, transcribed, and de-identified for analysis. The complete interview guides are provided as Supplementary Material (Patient, Organizational Stakeholder, Primary Care Provider, and Physical Therapist Interviews).

### 2.4 Data analysis

Qualitative data were analyzed using a structured thematic approach. Credibility, transferability, dependability, and confirmability were addressed using the trustworthiness parallel to quantitative research (Table 1) (38–41).

**Table 1:** Embedding methodological rigor using the trustworthiness parallel.

Methodological Rigor in Qualitative Research (38–41)	
Traditional Scientific Criteria for Quantitative Research	Trustworthiness Parallel for Qualitative Research
Internal validity	Credibility
External validity	Transferability
Reliability	Dependability
Objectivity	Confirmability

### 2.5 Analytic approach

The analytic process followed six key procedures to enhance methodological rigor (41).

1. Interview Guide Design: Interviews were conducted using a semi-structured-interview guide that featured open-ended, neutrally worded questions ordered from general to specific. The guide was standardized across participant roles to promote consistency in data collection and support dependability (39, 42, 43).
2. Codebook Development: A detailed codebook was created to define analytic codes, provide inclusion and exclusion boundaries, and offer representative examples. Independent double coding was followed by reflexive review of coding differences, with a third reviewer serving as mediator. Transcripts were recoded, and coding decisions were reconciled to establish the final codebook. This structure limited subjectivity in interpretation and supported confirmability (43–45).
3. Double Coding and Arbitration: Transcripts were independently coded by two team members, with discrepancies resolved by a third reviewer. This iterative comparison and reviewer triangulation supported credibility and inter-rater dependability (39, 46–48).
4. Reflexivity: Reflexive practices were embedded throughout data collection and analysis, including documentation of team discussions, coding decisions, and rationale for interpretation. This process minimized potential bias and contributed to confirmability (39, 45, 46, 49).
5. Thematic Recurrence: Patterns in data responses were assessed for recurrence and consistency across participants. The presence of repeated themes was used as evidence of the potential for findings to be relevant to similar settings (40, 44, 50).
6. Thematic Stratification: Final themes were stratified to identify barriers, mitigation strategies, and contextual insights relevant to telehealth physical therapy implementation. To preserve anonymity in this small, role-specific sample, site identifiers are excluded and quotations are presented without attribution (41, 44, 50).

## 3 Results

A thematic analysis of pre-implementation interviews yielded 10 overarching themes reflecting anticipated challenges, facilitators, and perceptions of telehealth, stratified by participant role (patients, providers, stakeholders). Supplementary Table S1 presents detailed supporting quotations for each theme across the three participant groups. Data were stratified by interviewee group type.

### 3.1 Patients

Patients consistently emphasized the anticipated value of physical therapy, describing it as likely to help improve pain and physical function. One patient shared, “*..it was very helpful with the different exercises*,” reflecting an expectation that guided therapy would offer tangible benefits. In addition to recognizing the potential impact of physical therapy, patients also expressed an awareness of its potential usefulness if delivered through telehealth. Several indicated appreciation for the opportunity to access care remotely. As one patient succinctly noted, when asked if telehealth would contribute to improving their access to care, “*Absolutely, yeah.*”

At the same time, patients anticipated barriers to access, particularly in relation to their home environments and comfort with telehealth technology. Some described expected challenges to performing exercises because of limited space or lack of equipment. One patient explained, “*..if my physical therapy required me to use equipment at home, that would be difficult,”* accentuating how physical context might influence the feasibility and consistency of at-home care delivery using telehealth.

### 3.2 Stakeholders

Stakeholders highlighted the anticipated availability of telehealth as a strength of the implementation, particularly given recent COVID-19 pandemic expansion efforts. Many described telehealth as having the potential to increase the reach of specialty care, especially for patients in remote rural and underserved areas. One stakeholder remarked, “*On a whole, we’re focused on trying to expand telehealth services*,” reflecting a forward-looking effort to build infrastructure and care delivery capacity.

In addition to capacity, stakeholders pointed to anticipated issues with access to technology, including broadband and digital devices. This concern is especially pronounced in rural areas, where infrastructure gaps persist. As one stakeholder noted, “*We’re [locally] really focused on broadband expansion,”* noting that consistent broadband connectivity would be a critical factor in the successful delivery of virtual, remote care.

Stakeholders also anticipated age-related concerns and potential differences in the acceptance of telehealth among older adults compared with younger patients. They expected that unfamiliarity with digital tools or a preference for in-person care could make adoption more difficult in this group. As one interviewee explained, “*..a lot of older individuals struggle with telehealth acceptance*,” but they suggested that tailored support strategies are needed to improve acceptance among older populations. The age-related theme presented here and in Supplementary Table S1 reflects participant perceptions of potential implementation barriers; participant ages were not collected, and no comparisons between age groups were conducted.

### 3.3 Providers

Providers identified a number of strategies they expected to use to support patients with chronic LBP via telehealth. One planned to recommend additional therapies or self-care techniques beyond physical therapy, such as posture correction and daily movement habits. One provider explained, “*I do have some home good back hygiene handouts,*” illustrating their intention to equip patients with tools for ongoing self-care.

Providers also emphasized the anticipated importance of patient education as part of the telehealth model. They expected that educating patients about chronic pain and offering guidance on exercises would help patients manage their LBP condition more independently. One provider noted*, “If the patient is having chronic back pain, there are certain exercises I advise,”* signaling a proactive approach to virtual education and empowerment.

Finally, providers strongly endorsed physical therapy as a central treatment modality for LBP. One described it as a “*favorite tool,”* highlighting the expectation that physical therapy, when adapted for telehealth, would be a great alternative to opioids for musculoskeletal pain. This endorsement also extended to the broader significance of telehealth and its role in improving access to specialty care, such as physical therapy treatment, among rural communities. This account reflects a provider's expectation about the role of physical therapy; changes in opioid use or prescribing were not assessed in these interviews.

### 3.4 Facilitators to implementation grouped by category and recurrence of theme

#### 3.4.1 Broadband and infrastructure expansion

Stakeholders emphasized that improving broadband infrastructure was expected to be a key facilitator of expanding access to telehealth, particularly for rural and underserved communities; these investments were viewed as necessary to support virtual care delivery. These data also reflected expectations that the current national investment in reliable broadband connectivity would be essential for telehealth, as it improves access to specialty care across geographically dispersed rural areas in general.

#### 3.4.2 Patient education and self-management

Providers identified patient education as an important strategy to support self-management through telehealth. They expected that delivering educational resources, especially for chronic conditions such as LBP, would allow patients to practice exercises at home with real-time or follow-up guidance. These data highlighted the potential utility of telehealth in reinforcing patient-led pain management.

#### 3.4.3 Flexibility and convenience of telehealth

Both patients and providers described flexibility and convenience as anticipated strengths of telehealth delivery. Patients expected that attending therapy sessions from home would reduce logistical barriers such as transportation and time away from work or caregiving; its convenience was seen as a path to improve their consistency with physical therapy attendance. Providers correspondingly noted that telehealth was expected to simplify appointment scheduling, increasing the likelihood that patients would complete their physical therapy sessions as planned.

#### 3.4.4 Decreasing the number of barriers to care access

Stakeholders highlighted that telehealth was expected to decrease the number of barriers to accessing initial consultations, especially for individuals in remote areas. They anticipated that virtual appointments would allow for earlier evaluations and referrals, helping patients begin physical therapy treatments for LBP more expeditiously. This early entry point was perceived as a key opportunity to strengthen a connection with specialty services that may not be locally available as well as a great opportunity to manage LBP in its earlier stages.

### 3.5 Barriers to implementation grouped by category and recurrence of theme

#### 3.5.1 Technology access and reliability

Stakeholders and patients identified technology access and internet reliability as expected barriers to effective telehealth implementation. Stakeholders emphasized that limited broadband in rural areas could prevent patients from engaging consistently in virtual sessions, indicating that infrastructure limitations were top of mind heading into implementation. Patients similarly expected to face challenges related to access to electronic devices or stable internet connectivity, which may impact the feasibility of remote physical therapy.

#### 3.5.2 Physical environment and equipment

Patients raised concerns about the physical requirements for home-based therapy, including the need for adequate space and equipment. Several patients described anticipated difficulties completing exercises in their home environments due to these limitations. This potential for environmental barriers could reduce the adherence to or effectiveness of therapy.

#### 3.5.3 Acceptance of and familiarity with telehealth

Stakeholders expected that telehealth acceptance would vary, particularly among older adults. They anticipated that some patients would be uncomfortable using the technology or prefer in-person interactions. These data reflected broader concerns that digital unfamiliarity could reduce engagement and uptake among certain populations.

#### 3.5.4 Therapeutic bond

Patients and providers both expressed concerns that building a strong therapeutic alliance between them could be more difficult in a virtual setting. Patients anticipated that it could be harder to communicate their needs or feel fully understood without being in the same room as their provider. Providers similarly questioned whether they would be able to establish trust and rapport through video alone. One patient shared, “*It's just different than being in the room with them; it's harder to connect*,” stressing how the remote modality of care delivery could negatively affect the interpersonal aspects of care.

### 3.6 Additional considerations for implementation grouped by recurrence of theme

#### 3.6.1 Team and collaboration dynamics

The data revealed noteworthy findings on team dynamics and collaboration. Stakeholders highlighted the importance of interdisciplinary collaboration in telehealth, emphasizing that coordination between healthcare providers, information technology (IT) specialists, and community organizations would be essential to the expansion of telehealth access and maintenance of quality care. One stakeholder specifically remarked on efforts to bring together “…*health departments and local providers..*” to coordinate resources and support. Both providers and stakeholders noted communication challenges within teams in telehealth settings, particularly when it came to coordinating patient care remotely. Providers described experiencing delays in communication and information sharing, which could be detrimental to patient outcomes and the timeliness of care. Stakeholders in rural and underserved areas also mentioned the lack of dedicated telehealth support teams, which they believed could help streamline service delivery and address technological barriers for patients.

Training and skill development was identified as another key component of successful rural telehealth implementation. Stakeholders emphasized the need for ongoing training, not only for providers, but also for administrative and technical staff who support telehealth operations. Some described a need to implement regular training sessions to improve staff confidence and enhance patient interaction and understanding in virtual settings.

Participants also discussed how telehealth presents a new need for role flexibility and expanded responsibilities as it evolves. Providers reported taking on additional administrative tasks in existing telehealth consultations, such as helping patients troubleshoot minor technical issues, while IT staff were now expected to become more familiar with healthcare-specific workflows. Several stakeholders noted an increased workload and additional responsibilities for support staff in scheduling and managing telehealth appointments, reflecting a shift in traditional roles, with newly added tasks to meet telehealth demands.

Finally, community partnerships were viewed as an extension of the care team, which is a common sentiment in rural health improvement efforts. Stakeholders emphasized collaborating with local organizations, such as libraries and community centers, to provide computers, internet access, and technical support for patients to learn about technology and be trained on using technology, especially in settings where socioeconomic barriers limit individual ownership of computers, familiarity with other digital tools, or both. This approach was seen as a practical way to leverage existing community infrastructure to facilitate broader familiarity with technology and psychological acceptance of remote telehealth.

## 4 Discussion

Qualitative data collection and analysis were integral to were integral to the development of the protocol for the ARBOR-Telehealth study preparation, as the data offered an understanding of implementation dynamics that would not have emerged through analysis of quantitative data alone. Findings from this baseline evaluation contributed to a more complete understanding of contextual factors of rurality, health service access, perceptions of telemedicine, and engagement in physical therapy activities. Qualitative input in randomized clinical trial development remains underused, with a dearth of detailed practical accounts showing how it informs trial planning and implementation (51). Explicitly documenting the application of the trustworthiness parallel provides an applied example that builds on foundational methodological literature and can inform subsequent studies (40, 41). Our findings align with rural implementation research highlighting technological infrastructure, staff capabilities, and communication (20). The collective perspectives informed planning with patients and healthcare systems for the parent ARBOR-Telehealth randomized clinical trial, described in its published protocol as among the first to examine telerehabilitation in a rural population (23).

Across patients, stakeholders, and providers, the data revealed a shared recognition of telehealth as a valuable addition to care, particularly in contexts such as rural communities, where physical access to services is limited. At the same time, several persistent challenges emerged, including limited access to, and familiarity with, necessary technology (i.e., broadband, computers, tablets) and the anticipated difficulty older adults may face in adapting to virtual platforms. The importance of patient education was a recurring theme among providers and stakeholders, who emphasized its role in helping individuals manage chronic pain and navigate their care more independently. Reported barriers to implementation included constraints within patients’ physical environments, discomfort or unfamiliarity with telehealth, and concerns about whether meaningful therapeutic relationships could be built remotely. In contrast, facilitators identified by participants included ongoing improvements to broadband infrastructure, educational tools that support self-management, and the perceived convenience and accessibility of virtual consultations, especially as a means of initiating early care for LBP in rural areas.

The parent ARBOR-Telehealth randomized clinical trial study design (23) incorporated adaptations to address several of these challenges based on data from this analysis. In response to the identified need for patient education, patients were provided with educational materials tailored to support self-management of LBP. To address limited access to exercise equipment, physical therapy supplies were mailed to patients in advance of their sessions to support home-based participation. Patients were offered one-on-one technical assistance with internet connectivity, sign-on portal issues, and device-related difficulties to facilitate access to telehealth appointments. Further, protocols for physical therapist training and standardized communication approaches were developed and distributed in response to concerns about therapeutic relationships, with the aim of preserving the therapeutic bond in a virtual setting (23).

Our data also revealed several surprising team and collaboration considerations influencing the success of telehealth implementation. Whereas interdisciplinary collaboration is central to operationalizing telehealth, participants identified challenges with communication, coordination and additional work, suggesting adaptations to traditional workflows might be required, as both clinical and non-clinical staff are expected to support telehealth tasks that extend beyond their typical responsibilities. Training and clarity of responsibility emerged as necessary components with stakeholders and providers anticipating that ongoing support would be critical to navigating both new technologies and evolving care processes among themselves and with patients. Additionally, partnerships with community organizations were seen as a means of augmenting team capacity, particularly in rural areas where access to technology is especially limited, by providing supplemental training and tapping into existing local infrastructure to enable patient participation. The prominence of team dynamics and coordination may reflect the complementary perspectives of participants involved in receiving, delivering, and organizing care within the participating rural healthcare system. The study's pre-implementation focus may also explain the attention given to anticipated workflow and support needs at TidalHealth.

There are several key strengths to the analyses. First, the data revealed the interconnectedness of concerns among study patients, stakeholders, physical therapists, and providers in the implementation. Second, findings reflected first-hand participant experiences and viewpoints that informed implementation planning. Third, the interview format had the flexibility to follow and explore tangential information that would otherwise be unaccounted for in quantitative analyses alone. Fourth, the data offered contextual guidance for designing implementation strategies responsive to concerns raised by interviewees, including messaging and support tailored to participant roles (i.e., peer perspectives for both physical therapists and patients); these insights can inform similar studies and their implementation in rural settings.

A limitation of the study was the potential for the results to be influenced by social desirability bias and selection bias. Additionally, the project-specific limitation of a small interviewee sample size limits generalizability of the data. The absence of participant demographic information further limits assessment of the transferability of these perspectives. The findings also cannot imply causality. Further, there is no internal consistency reliability because the data were not collected at different implementation timepoints but, rather, as baseline data to guide and optimize implementation efforts and mitigate potential barriers to the intervention's success. To assuage the limitations of the biases noted, methodological rigor using the trustworthiness parallel to quantitative research (40) was embedded in every component of the qualitative evaluation design, data collection, and analytic approach.

## 5 Conclusion

These data revealed key rural-specific contextual factors for shaping the successful implementation of telehealth-delivered physical therapy in rural settings. Through baseline interviews with patients, providers, and stakeholders, participants identified anticipated technological, environmental, and interpersonal barriers and facilitators to the adoption and delivery of telehealth services. The findings highlighted both challenges that might affect patient engagement and actionable strategies to mitigate those challenges. By anticipating areas where role flexibility, interdisciplinary collaboration, and local infrastructure support is needed, rural telehealth providers can improve feasibility and engagement with remote physical therapy.

This work contributes to ongoing national efforts by the NIH HEAL initiative to improve pain management and reduce reliance on opioids in communities disproportionately affected by the overdose crisis, such as rural communities (22, 23). Telehealth has become an increasingly vital and relevant tool for bridging geographic and resource gaps in expanding access to specialty healthcare, such as remote physical therapy (2). This is especially relevant as rural populations continue to face structural and logistical barriers to accessing nonpharmacologic treatment options for chronic pain, such as LBP (11, 15, 16), and scalable models for delivering physical therapy remain limited (52, 53). As the ARBOR Telehealth study (26) is the first of its kind in rural settings, this qualitative evaluation provides early, context-specific insights into the feasibility of delivering guideline-based physical therapy through telehealth. Collectively, these perspectives informed pre-implementation planning for the ARBOR-Telehealth randomized clinical trial, in which clinical effectiveness and opioid-related outcomes are being evaluated (23). These findings provide contextual guidance for the design and implementation of telehealth-delivered physical therapy interventions aimed at strengthening long-term pain management capacity and ultimately reducing opioid reliance in rural communities in the United States.

## Data Availability

The original contributions presented in the study are included in the article/Supplementary Material, further inquiries can be directed to the corresponding author.
