# Supplementary material for: Implementation of rural telehealth physical therapy for chronic low back pain: qualitative findings informing a randomized clinical trial aimed at decreasing opioid use
Source: Front Rehabil Sci. 2026 Oct 27;7:1951507. doi: 10.3389/fresc.2026.1951507 (PMC13748409; doi:10.3389/fresc.2026.1951507)
Supplement: Supplementary file 1 [file Datasheet1.pdf]

Supplementary Data

Supplementary Table 1: Overarching Themes and Supporting Quotations Across Interviewee Roles from the ARBOR Telehealth Study Data

Overarching Theme	Description of Theme	Supporting Patient Quote	Supporting Stakeholder Quote	Supporting Provider Quote
Access to care	Challenges to the ability to obtain timely healthcare services	*"I'm still recovering, but not having to travel on days that I'm really hurting [is useful]…some of the places are anywhere from half an hour, 45 minutes to an hour and a half, to 2 hours…so video visits are quite helpful in the fact that that's one less time I have to get in the car and suffer the trip."*	*"...another challenge is no-shows…for just visits in general [due to transportation]…for folks that really needed services. Folks were below the poverty level… [and] know they needed to utilize services. And the sliding-fee-scale [patients]…were the highest no-show rates."*	*"For those in rural areas, sometimes it's hard to even get a specialist to look at their case."*
Access to technology	Availability and accessibility of technology required for telehealth	*"If my physical therapy required me to use equipment at home, that would be difficult [with space]."*	*"Broadband is essential for telehealth, especially in rural areas."*	*"There are patients where the internet is so spotty it's hard to get through a full session."*

**Overarching Theme**	**Description of Theme**	**Supporting Patient Quote**	**Supporting Stakeholder Quote**	**Supporting Provider Quote**
Age-related differences	Differences in telehealth acceptance or experience based on age	*"...you would think that a majority of patients have access, but it's not always the case…for older patients."*	*"Older individuals struggled a lot more to adopt telehealth compared to younger patients [during the COVID-19 pandemic]."*	*"I noticed that my older patients had more trouble connecting to the session than younger ones."*
Availability of telehealth	Presence and accessibility of telehealth services in systems	*"I was glad telehealth was available, otherwise I would have had to travel far for my appointment."*	*"The majority of our providers are now offering telehealth, especially post-COVID."*	*"It's great that telehealth is an option now. I can see my patients without them coming in every time."*
Awareness of usefulness	Realization of the benefits and effectiveness of telehealth or physical therapy	*"Absolutely, it helped me keep up with my therapy without missing anything."*	*"Many patients didn't realize how convenient telehealth could be until they tried it."*	*"I think more patients are [now] understanding that telehealth can be just as effective."*
Broadband internet connectivity	Availability and stability of internet necessary for telehealth	*"Sometimes the connection would drop, and it made it hard to understand what they [the providers] were saying."*	*"Broadband is essential for telehealth, especially in rural areas."*	*"...there are some areas…pretty significant areas, that don't have internet access. They have, like ...some [individual] satellite. I guess that does not work that well, so yeah, I think that [internet connectivity] is a concern."*

**Overarching Theme**	**Description of Theme**	**Supporting Patient Quote**	**Supporting Stakeholder Quote**	**Supporting Provider Quote**
Comparisons of telehealth to in-person care	Comparative perceptions between telehealth and traditional in-person care	*"It's helpful, but I still miss being able to see my therapist in person."*	*"Some people still prefer in-person, but telehealth is a viable option."*	*"Telehealth is great for convenience, but there are still things you can't do without being there."*
Exercise equipment and space	Access to adequate space and equipment for telehealth physical therapy sessions	*"I don't have the space at home for the exercises they ask me to do."*	*"Not all patients have room at home to follow-through with their therapy."*	*"Some exercises require equipment that patients don't have, which can be a limitation."*
Lack of acceptance of telehealth	Reluctance or discomfort using telehealth, often due to preference for in-person care	*"I just don't feel comfortable with it; I'd rather go in person."*	*"There's still resistance to telehealth, especially among older adults."*	*"Some of my patients just don't like telehealth and would rather wait for an in-person slot."*
Therapeutic bond	The bond and trust between patient and provider and its effect on care delivery	*"It's just different than being in the room with them; it's harder to connect."*	*"The therapeutic alliance can suffer when it's virtual, especially for new patients."*	*"I feel like I can't build the same connection over a screen as I would in person."*
