# Supplementary material for: Implementation of rural telehealth physical therapy for chronic low back pain: qualitative findings informing a randomized clinical trial aimed at decreasing opioid use
Source: Front Rehabil Sci. 2026 Oct 27;7:1951507. doi: 10.3389/fresc.2026.1951507 (PMC13748409; doi:10.3389/fresc.2026.1951507)
Supplement: Supplementary file 2 [file Datasheet2.pdf]

ARBOR TELEHEALTH

Implementing Telerehabilitation for Patients with Chronic Low Back Pain in Rural Maryland

We want to find the best way to help people who live in rural communities and have low back pain. We think using video calls on the Internet might be a good way to give them physical therapy. We're planning a research study to test this idea with people in rural Maryland who have chronic low back pain. Our goal is to make it easier for people to get help for their back pain and to make them feel better without needing strong pain pills.

We'd like you to talk to us about your experience with back pain. There are no right or wrong answers. Your thoughts will help us plan our study better.

Thank you for helping us make healthcare better for people who live far away from cities. Below are some questions we'll ask during our chat. Take a look and jot down any thoughts you have before we talk.

1. Have you ever received physical therapy for low back pain?
    a. Yes
    b. No

    - *Interviewee Probe, **if Yes**: did you find physical therapy to be helpful in managing your condition?*
        i. *Yes – very helpful.*
        ii. *Yes – somewhat helpful.*
        iii. *No – not helpful.*
        iv. *Neutral – neither yes nor no.*

2. Have you received physical therapy for any other conditions in the past?
    a. Yes
    b. No

    - *Interviewee Probe, **if Yes**: did you find physical therapy to be helpful in managing your condition?*
        i. *Yes – very helpful.*
        ii. *Yes – somewhat helpful.*
        iii. *No – not helpful.*
        iv. *Neutral – neither yes nor no.*

3. Have you ever received physical therapy using telehealth visits?
    a. Yes
    b. No

4. Have you received healthcare services (such as primary care) other than physical therapy using telehealth visits?
    a. Yes
    b. No

5. Can you think of barriers that would impact your ability to receive physical therapy using telehealth video visits?

    [Open response]

6. Do you have recommendations on how our team could help you to overcome these barriers?

    [Open response]

7. What are your thoughts on the quality of care a physical therapist can provide using telehealth video visits?

    - *Interviewer Prompt: Quality of care is, for example, how well the therapist helps you and how much they listen to you.*

    [Open response]

8. Do you have suggestions for how we can improve your or experience in receiving physical therapy via telehealth video visits?

    [Open response]

Implementing Telerehabilitation for Patients Living in Rural Maryland (Organizational Stakeholder Interview)

We want to find the best way to help people who live in rural communities and have low back pain. We think using video calls on the Internet might be a good way to give them physical therapy. We're planning a research study to test this idea with people in rural Maryland who have chronic low back pain. Our goal is to make it easier for people to get help for their back pain and to make them feel better without needing strong pain pills.

We'd like you to talk to us about your experience in an organization that helps people in rural communities with their health conditions, including back pain. There are no right or wrong answers. Your thoughts will help us plan our study better.

Thank you for helping us make healthcare better for people who live in rural communities. Below are some questions we'll ask during our chat. Take a look and jot down any thoughts you have before we talk.

Interview Questions

1. What is your role in your organization?

2. Could you share how your organization interacts with
    a. Health systems who have patients with chronic low back pain?

    [Open response]

    b. Providers who care for patients with chronic low back pain?

    [Open response]

c. Patients themselves with chronic low back pain?

[Open response]

3. How has your organization prioritized the burden of chronic low back pain?

[Open response]

4. What types of healthcare services do your members typically provide for patients with low back pain?

[Open response]

5. What are some barriers that make it difficult for health systems or providers to offer telehealth physical therapy for patients with low back pain? Please elaborate.

    - *Interviewer Prompt: How have health systems or providers addressed these barriers?*

[Open response]

- What are some barriers that make it difficult for patients to access telehealth physical therapy? Please elaborate.

    - *Interviewer Prompt: How can organizations support patients to address these barriers?*

[Open response]

- What are your thoughts on the use of telehealth physical therapy for patients with chronic low back pain?

    - *Interviewer Prompt: How do you think that telehealth services could support patient with chronic low back pain?*

  [Open response]

- Do you have suggestions on how we could make this study more relevant to:
    a. Patients with chronic low back pain who live in rural communities?

    [Open response]

    b. To the health systems and providers that care for these patients?

    [Open response]

    c. To the health systems and providers that care these patients?

    [Open response]

Implementing Telerehabilitation for Patients Living in Rural Maryland - Primary Care Provider (PCP) Interview

We want to find the best way to help people who live in rural communities and have low back pain. We think using video calls on the Internet might be a good way to give them physical therapy. We're planning a research study to test this idea with people in rural Maryland who have chronic low back pain. Our goal is to make it easier for people to get help for their back pain and to make them feel better without needing strong pain pills.

We'd like you to talk to us about your experience as a clinician who cares for people in rural communities, including those living with back pain. There are no right or wrong answers. Your thoughts will help us plan our study better.

Thank you for helping us make healthcare better for people who live in rural communities. Below are some questions we'll ask during our chat. Take a look and jot down any thoughts you have before we talk.

Interview Questions

1. How often do you see patients with low back pain in clinic?
    a. Daily
    b. Weekly
    c. Monthly
    d. Less than monthly

2. What types of <u>healthcare services</u> do you typically recommend and/or prescribe for patients with low back pain presenting without red flag symptoms?

    [Open response]

3. What type of recommendations do you make for these patients except for what types of providers they should see (e.g., behavioral modifications, diet, medication)?

   [Open response]

4. How often (in percentage) do you recommend physical therapy for your patients with low back pain?

   [Numerical % response]

5. Do you think there are barriers that make it challenging for your patients to attend physical therapy? Please elaborate on your answers.

   [Open response]

6. What do you think about using telehealth physical therapy for patients with chronic low back pain?

   [Open response]

7. Do you have suggestions on how we could make this study more relevant to your patients with chronic low back pain?

   [Open response]

Implementing Telerehabilitation for Patients Living in Rural Maryland - Physical Therapist (PT) Interview

We want to find the best way to help people who live in rural communities and have low back pain. We think using video calls on the Internet might be a good way to give them physical therapy. We're planning a research study to test this idea with people in rural Maryland who have chronic low back pain. Our goal is to make it easier for people to get help for their back pain and to make them feel better without needing strong pain pills.

We'd like you to talk to us about your experience as a physical therapist who provides care for people in rural communities who are living with back pain. There are no right or wrong answers. Your thoughts will help us plan our study better.

Thank you for helping us make healthcare better for people who live in rural communities. Below are some questions we'll ask during our chat. Take a look and jot down any thoughts you have before we talk.

Interview Questions

1. Have you ever provided physical therapy using real-time video visits?
    a. Yes
    b. No

2. *Interviewer Probe: If yes, how many physical therapy video visits have you completed? Please provide an estimate.*
    c. Less than 10 video visits
    d. 10-50 video visits
    e. 50-100 video visits
    f. Over 100 video visits

2. When was the last time you conducted a physical therapy video visit?
    a. Within the past 1-month
    b. Within the past 6 months
    c. Within the past 12 months
    d. More than 12 months ago

3. Are there barriers you think might influence a patient's ability to receive physical therapy using real-time video visits? Please elaborate.*[Open response]*

4. Do you have suggestions on how we might help patients overcome these barriers?

    [Open response]

5. Are there barriers you think might impact your ability to provide quality care for patients with chronic low back pain via real-time video visits?

    [Open response]

6. Do you have recommendations for how we could help you to provide high quality care to patients with chronic low back pain via video visits?

    [Open response]
